# Polyvinyl-Alcohol-Modified Calcium Sulphoaluminate Cement Repair Mortar: Hydration and Properties

**DOI:** 10.3390/ma14247834

**Published:** 2021-12-17

**Authors:** Yongjie Bian, Yongbo Huang, Fuxin Li, Dong Dong, Honggen Zhao, Piqi Zhao, Lingchao Lu

**Affiliations:** 1 Shandong Provincial Key Lab of Preparation and Measurement of Building Materials, University of Jinan, Jinan 250022, China; b1403662721@126.com (Y.B.); dong03092@163.com (D.D.); zhao_honggen@163.com (H.Z.); mse_zhaopq@ujn.edu.cn (P.Z.); lingchao_lu@163.com (L.L.); 2 State Key Laboratory of Green Building Materials, China Building Materials Academy, Beijing 100024, China; 3 China United Cement Pingyi Co., LTD, Pingyi 273300, China; 18853962680@163.com

**Keywords:** polyvinyl alcohol, calcium sulphoaluminate cement, repair mortar, hydration and properties

## Abstract

Polyvinyl alcohol (PVA) and calcium sulphoaluminate (CSA) cement were used to prepare repair mortar for the restoration of the walls of a building built with bricks. The preparation, hydration, and properties of the PVA-modified CSA cement repair mortar were studied. Besides this, the mechanism by which PVA improves the bonding strength is also discussed. The results demonstrate that PVA prolongs the setting time of CSA cement, which is ascribed to PVA inhibiting the dissolution of C_4_A_3_$ (4CaO·3Al_2_O_3_·SO_3_) and the precipitation of AFt (3CaO·Al_2_O_3_·3CaSO_4_·26H_2_O) within the hydration age of 0~60 min. PVA lowers the mechanical strength of CSA cement repair mortar at the hydration age of 6 h. After 6 h, the mechanical strength is improved. PVA could also improve the bonding strength between CSA repair mortar and bricks. This is mainly ascribed to the Al ions in both the hydration products of CSA cement and the clay bricks reacting with the hydroxyl group of PVA and forming the chemical bond C-O-Al. Therefore, a tighter combination between CSA cement repair mortar and the clay bricks forms, thereby improving the bonding strength.

## 1. Introduction

Due to their direct exposure to the natural environment, the buildings in service are inevitably subjected to external mechanical forces and environmental corrosion. With prolonged service life, structural damage arises, which poses a great threat to personal safety and shortens the service life [1,2,3,4]. Therefore, buildings with structural damage are in urgent need of repair.

In China, most of the walls of residential buildings were built with bricks in the twentieth century. Because of the porous structure of the bricks, the wall structure exhibits strong water absorption, which makes repair mortars easily fall off due to water loss, leading to the failure of repair. To achieve the goal of perfect restoration of a wall structure built with bricks, the repair mortar should have good water retention and high bonding strength. Additionally, to shorten the duration of repair works, the repair mortars must also have the rapid hardening property [5,6,7].

To improve the bonding strength between the repair mortar and the old substrate, polymers such as polyvinyl alcohol [8,9], ethylene-vinyl acetate [10,11], methyl cellulose [12,13], epoxy resin [14,15], and styrene butadiene rubber [16] have been introduced into repair materials. These polymers could also improve the working performance [17] and mechanical properties [18,19,20] of mortar, reduce the porosity [3,21], and improve the impermeability [22,23] and durability [24,25]. PVA exhibits appropriate water absorption [8,26]. Therefore, repair mortar prepared with PVA could have good water retention performance, which makes it more available for the restoration of damaged structures of walls built with bricks.

Previous studies found that the carboxyl group on the polymer branch chain hydrolyzes in the Portland cement hydration system, and the generated acetic acid reacts with Ca^2+^ in the pore solution to produce Ca(CH_3_COO)_2_. At the same time, the hydroxyl group could combine with Ca in the hydration product to form a C-O-Ca chemical bond [27,28]. At present, PVA has been widely applied in cement-based materials [8,29,30]. A similar reaction occurs in calcium aluminate cement [31,32] and magnesium phosphate cement [26], forming the chemical bonds C-O-Al and C-O-Mg, respectively. These chemical bonds could join the polymer film and the hydration products, which leads to a tighter combination of the hydration products and improves the mechanical strength [33,34,35]. For CSA cement, the hydration products contain Ca and Al ions, which may combine with the hydroxyl group in PVA and form the chemical bonds C-O-Ca and C-O-Al, respectively. This contributes to the improvement of the mechanical strength.

Compared with Portland cement, calcium sulphoaluminate (CSA) cement shows relatively faster hardening and higher early age strength. Besides this, the drying shrinkage of CSA cement is much lower. All of these make it more suitable for structural repair [21,26,36].

To satisfy the requirements of repair materials for the restoration of wall structures built with bricks, PVA was used herein as a modifier of CSA cement to prepare repair mortar. The preparation, hydration, and properties of the PVA-modified CSA cement repair mortar were studied. In addition, the mechanism by which PVA improves the bonding strength is discussed.

## 2. Experimental Procedures

### 2.1. Materials

**Figure 1:**
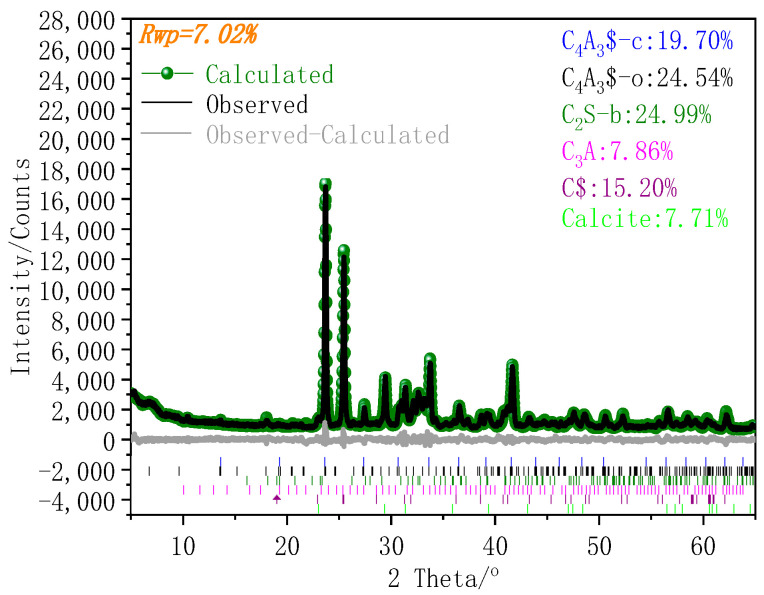
X-ray analysis result of CSA cement.

**Figure 2:**
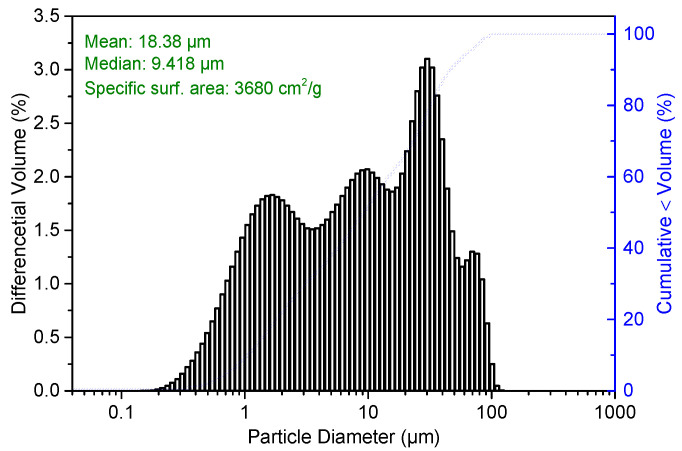
Particle size distribution of CSA cement.

CSA cement (42.5R, produced by China United Cement Co., Ltd., Jining, China) and sand with diameter below 4.75 mm were used to prepare the cement repair mortar. Polycarboxylate superplasticizer was used to reduce the water demand of the CSA cement repair mortar. Tributyl phosphate and citric acid were used as a defoamer and retarder, respectively. The chemical composition and phase composition of CSA are shown in Table 1 and Figure 1, respectively. The particle size distribution of CSA cement is shown in Figure 2. Since the sand was not standard sand, the physical properties of the sand were measured according to Chinese standard GB/T 14684-2011, and the results are shown in Table 2.

PVA produced by Shanghai Yingjia Industrial Development Co., Ltd., Shanghai, China with a low degree of polymerization was used as a modifier; its physical and chemical properties are shown in Table 3.

**Figure 3:**
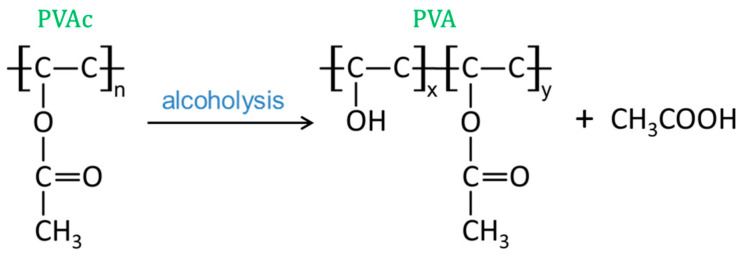
Alcoholysis reaction of PVAc.

**Figure 4:**
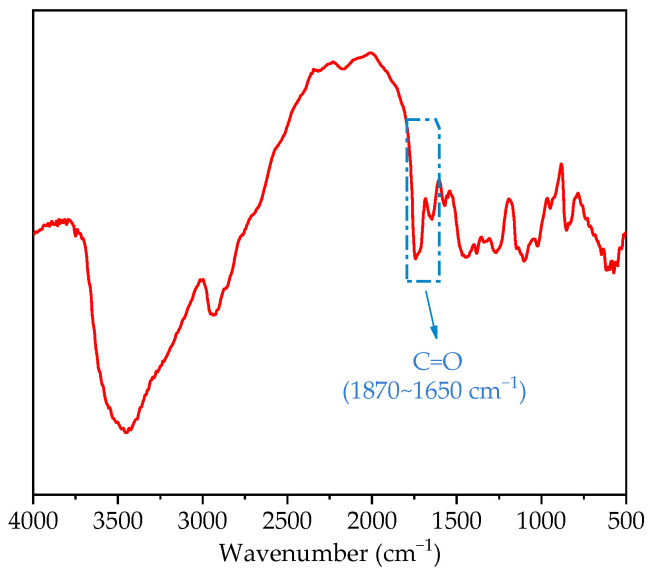
FITR spectrum of the PVA powder.

PVA is produced from polyvinyl acetate through an alcoholysis reaction (as shown in Figure 3), where x/(x + y) is the alcoholysis degree. In the process of alcoholysis, the acetate (-OOCCH_3_) in polyvinyl acetate (PVAc) is replaced by a hydroxyl group. The alcoholysis degree of the PVA used in this study was 87.0~89.0%. FTIR results confirmed the existence of C=O in the PVA powder (Figure 4).

### 2.2. Sample Preparation

CSA cement repair mortar samples with dimensions of 40 mm × 40 mm × 160 mm were prepared according to Chinese standard GB/T 17671-1999 to test the compressive and flexural strength. After 6 h, the cement mortars were de-molded, and the samples were cured in a standard chamber with a temperature of 20 ± 2 °C and a relative humidity of 90 ± 5%. The hydration of the CSA mortars was terminated by immersing the samples in absolute ethanol for 24 h. Cement pastes were prepared with a water-to-cement ratio of 0.35. After curing in the standard chamber with a temperature of 20 ± 2 °C and a relative humidity of 90 ± 5%, the hydration of CSA cement paste samples was terminated by immersing them in absolute ethanol for 24 h.

### 2.3. Test Methods

#### 2.3.1. Compressive and Flexural Strength

At the hydration ages of 6 h, 1 d, 3 d, 7 d, and 28 d, the compressive and flexural strength were tested using a universal testing machine (MTS CMT 5504, Eden Prairie, MN, USA).

#### 2.3.2. Tensile Bonding Strength

The tensile bonding strength between the CSA cement repair mortar and the bricks was determined according to Chinese standard JGJ/T 70-2009 using a universal testing machine MTS CMT 5504. The dimensions of the CSA cement repair mortar test cubes were 40 mm × 40 mm × 10 mm. To avoid insufficient hydration of CSA cement caused by the water absorption of the bricks, the bricks were immersed in water for 5 min. After the bricks were taken out of the water, the water on the surface was removed using a dry towel.

#### 2.3.3. Fluidity

The fluidity of the CSA cement repair mortar was determined according to Chinese standard GB/T 2419-2005.

#### 2.3.4. Setting Time

The setting times of the cement paste were tested according to Chinese standard GB/T 1346-2011.

#### 2.3.5. Particle Size Distribution

After full dispersion in ethanol, the particle size distribution of CSA cement was tested using a Mastersizer 3000.

#### 2.3.6. Chemical Composition

By pressing the cement powder at 1 MPa, a tablet was obtained. The chemical composition, expressed in oxides, of the CSA cement was determined by examining the tablet using an S8 TIGER X-ray fluorescence spectrometer.

#### 2.3.7. FTIR (Fourier Transform Infrared) Analysis

FTIR analysis was conducted using an infrared spectrometer (Nicolet 380, America) with a DGTS CsI detector, and 32 scans were recorded. The scans were taken in the mid-infrared region at frequencies of 4000 cm^−1^ to 500 cm^−1^, with a spectral resolution of 4 cm^−1^. To ensure the transmittance of the disc, it was prepared by mixing 0.1 mg PVA powder with 100 mg KBr. Then, the mixture was homogenized and pressed into discs at a pressure of 10 MPa.

#### 2.3.8. XRD (X-Ray Diffraction) Analysis

After grinding the samples to pass through a 75 μm sieve, the powder samples were subjected to XRD analysis. The measurement was conducted using an X-ray diffractometer (D8 ADVANCE, Bilerica, MA, USA) with Cu Kα radiation at a voltage of 40 kV and a current of 40 mA, with a step size of 0.02° and a scan speed of 5°/min. To determine the hydration products of CSA cement at 3 min, 5 min, 15 min, 30 min, and 60 min, measurements were conducted over the range of 7~14°. To determine the hydration products of CSA cement at 6 h, 1 d, and 3 d, measurements were conducted over the range of 5~55^°^. For CSA clinker, measurements were conducted over the range of 5~65° with a scan speed of 1°/min. The quantitative XRD (QXRD) method was adopted to determine the mineral composition of CSA cement using Topas 4.2 software.

#### 2.3.9. Thermal Analysis

After vacuum drying to remove the free water and milling to pass through a 75 μm sieve, cement paste powder was subjected to TG analysis. TG testing was conducted using a thermal analyzer (TGA/DSC1/1600HT, Mettler Toledo, Zurich, Switzerland) with a heating rate of 10 °C/min within the temperature range of 30~800 °C. The experimental atmosphere was Ar and the flow rate was 50 mL/min. The weight of each sample was 35 ± 5 mg.

#### 2.3.10. Hydration Heat

The hydration heat of PVA-modified CSA cement was measured using a calorimeter (TAM Air, TA Instrument, Newcastle, DE, USA) with an accuracy of ±20 μW and an operating temperature of 20 ± 0.2 °C. The heat flow was recorded every 24 s until 24 h had passed.

#### 2.3.11. Ion Concentration and pH of the Pore Solution and SI of Aft

The pore solution was obtained by centrifugation of the suspension (using ultrapure water with a resistance of 18.2 × 10^6^ Ω·cm) with a water-to-cement ratio of 2:1. The pH value of the pore solution was tested using an FE28 pH-meter (Mettler Toledo, Zurich, Switzerland). After acidization by nitric acid, the ion concentration of the pore solution was tested via inductively coupled plasma optical emission spectroscopy (PerkinElmer optima 8000, Waltham, MA, USA) at 25 °C.

The SI of AFt can be calculated using PHREEQC software via Equation (1) based on the measured Ca, S, and Al concentrations and the pH value of the pore solution. The SI of AFt was calculated according to Equation (1):

(1) $$\mathrm{SI}=\mathrm{lg}\frac{\mathrm{IAP}}{K_{\mathrm{sp}}}$$

where IAP and K_sp_ are the ion activity product and the solubility of AFt at 25 °C, respectively.

The lgK_sp_ of AFt at 25 °C is −44.90. The IAP of AFt was calculated according to Equation (2):

(2) $$\mathrm{IAP}={\left({\mathrm{Ca}}^{2+}\right)}^{6}{\left({\mathrm{AlO}}_{2}{}^{-}\right)}^{2}{\left({\mathrm{SO}}_{4}{}^{2-}\right)}^{3}{\left({\mathrm{OH}}^{-}\right)}^{4}$$

The ion activity was calculated according to Equation (3):

(3) $$a_{i}=c_{i}\gamma_{i}$$

where $a_{i}$ is the activity of ion i, $c_{i}$ is the concentration of ion i, and $\gamma_{i}$ is the activity coefficient of ion i.

The activity coefficient was calculated according to the extended Debye–Hückel equation (Equation (4)). It can be calculated using PHREEQC software.

(4) $$\log\gamma_{i}=\frac{-A_{\gamma }z_{i}^{2}\sqrt{I_{m}}}{1+\dot{a}B_{\gamma }\sqrt{I_{m}}}+b_{\gamma }I_{m}$$

#### 2.3.12. X-Ray Photoelectron Spectroscopy (XPS)

XPS analysis was carried out using an ESCALAB Xi+ from Thermo Scientific with monochromatic Al Kα radiation (300 W, 20 mA, 15 kV). The data were imported into Avantage software (version 5.9915) for manipulation and curve fitting. To compensate for the surface charge effects, the binding energies were calibrated using the C 1s hydrocarbon peak at 284.80 eV.

## 3. Results and Discussion

### 3.1. Composition of CSA Cement Repair Mortar

To achieve perfect mechanical interlocking between the CSA cement repair mortar and the bricks, good fluidity—which has close ties with the free water content—of the CSA cement repair mortar is required. The addition of PVA increases the viscosity of CSA cement. Besides this, the hydroxyl groups in PVA can form hydrogen bonds with water molecules, which absorbs the free water and lowers the fluidity of the CSA repair mortar. To ensure sufficient free water to maintain fluidity, polycarboxylate superplasticizer was introduced into the CSA repair mortar as a water-reducing agent.

The composition of the CSA cement repair mortar is shown in Table 4. With increasing PVA content, to maintain good fluidity, the polycarboxylate superplasticizer content was increased. When the PVA content reached 2.0%, the demand for polycarboxylate superplasticizer increased significantly, leading to a significant increase in the cost of the repair mortar. Therefore, the PVA content should be controlled within the range of 0~1.5%. Besides this, the early hydration of CSA cement has close ties with fluidity loss over time [21]. Therefore, 0.2% citric acid was introduced into the CSA cement mortar as a retarder. Under the combined action of polycarboxylate superplasticizer and citric acid, the fluidity of the CSA repair mortar can be controlled within the range of 169.5~170.5 mm, which can meet the requirements of structural repair.

Previous investigations found that the addition of polymer into cement mortar incurs an increase in the viscosity, which makes it difficult to eliminate air voids. Therefore, the porosity of the hardened cement increases [37,38]. To remove the air-entraining effect, tributyl phosphate (0.2 wt%) was added into the CSA cement repair mortar as a defoamer to remove the air voids.

### 3.2. Properties

**Figure 5:**
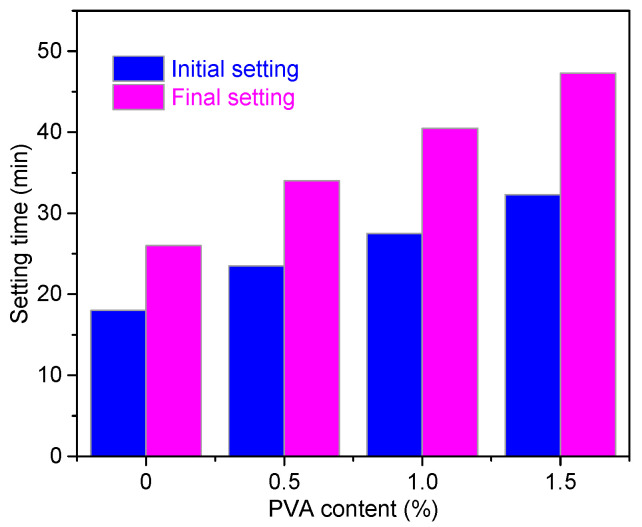
Setting times of CSA cement pastes with and without PVA.

For CSA cement, the setting time has close ties with the initial hydration of C_4_A_3_$ and the precipitation of the hydration products [39,40]. Figure 5 shows the initial and final setting times of CSA cement with different amounts of PVA. With increasing PVA content, both the initial and final setting times were obviously prolonged. This is probably due to the following reasons: (i) PVA molecules concentrate on the surface of CSA cement particles, which hinders the initial dissolution of the cement minerals and inhibits the early precipitation of the hydration products [26]. (ii) PVA contains a slight amount of residual acetic acid and polyvinyl acetate. Therefore, PVA lowers the pH value of the pore solution, which could also inhibit the early age precipitation of the hydration products.

Although 0.2% citric acid was added into the CSA cement to prolong the setting time, the initial setting time and final setting time for CSA cement without PVA were merely 10 min and 26 min, respectively, which are too short to satisfy the demands of structural repair. Compared with those of the Reference samples, the initial setting time and final setting time of CSA cement with 1.5% PVA increased by 79.4 and 81.9%, respectively, making it more suitable for structure repair.

**Figure 6:**
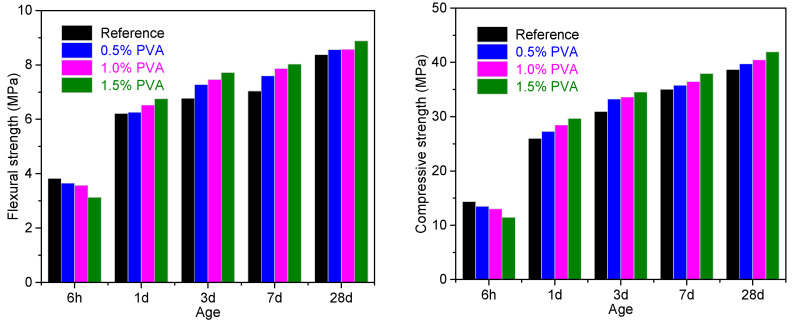
Flexural and compressive strength of the Reference mortar and the CSA cement mortars with 0.5% PVA, 1.0% PVA, and 1.5% PVA.

Figure 6 shows the compressive strength and flexural strength of the Reference samples and the CSA cement repair mortar samples with 0.5% PVA, 1.0% PVA, and 1.5% PVA. At the hydration age of 6 h, with increasing PVA content, both the compressive strength and flexural strength decreased. This is probably due to the following reasons: (i) PVA inhibits the hydration of C_4_A_3_$ within the hydration age of 6 h. (ii) At the hydration age of 6 h, PVA fails to form a film and is evenly distributed among the cement particles and cement hydration products, which weakens the bonding strength between the hydration products and thereby reduces the strength [33,37].

At the hydration ages of 1 d, 3 d, 7 d, and 28 d, both the compressive strength and flexural strength of CSA cement repair mortars with 0.5% PVA, 1.0% PVA, and 1.5% PVA exceeded those of the Reference. This is probably due to the following reasons [34,35]: (i) PVA forms a film that fills the cracks of the hardened CSA cement repair mortar, which lowers the porosity of the hardened CSA cement repair mortar and thereby improves mechanical properties. (ii) The PVA films distribute among the particles of the hydration products. The hydroxyl group in PVA could react with the ions of the hydration products, improving the linkage between the hydration products and thereby promoting the compressive strength and flexural strength [32].

**Figure 7:**
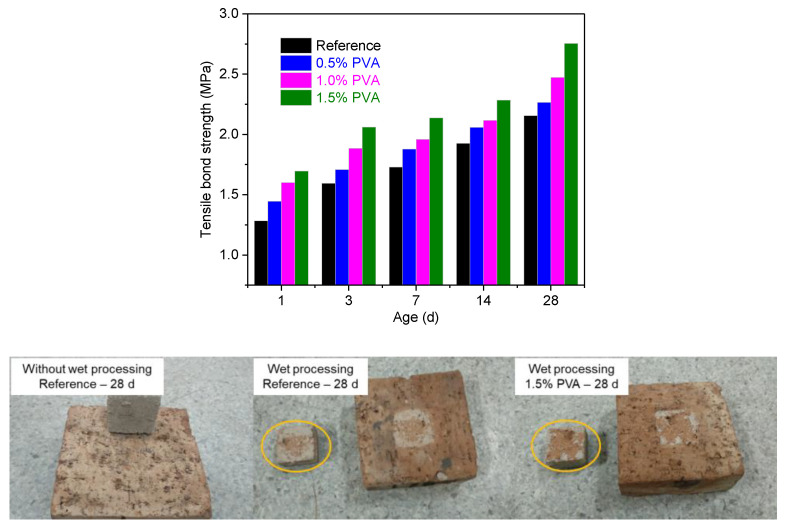
Bonding strength and fractured surface between the CSA cement repair mortars and the clay bricks.

The bonding strength between the CSA cement repair mortar and clay bricks was studied, and the result is shown in Figure 7. At all hydration ages (1 d, 3 d, 7 d, 14 d, and 28 d), the bonding strength increased with increasing PVA content. At the hydration age of 28 d, compared with that for the Reference sample, the bonding strength between the CSA cement mortar with 1.5% PVA and the brick increased by 35.47%. Therefore, it is concluded that PVA can enhance the connection between the CSA cement repair mortar and clay bricks. This is probably due to the hydroxyl group in PVA reacting with the ions in the CSA cement mortar and the bricks, which leads to a tight combination between them and thereby improves the bonding strength. Previous investigations also found that PVA could improve the bonding strength between Portland cement mortar and old concrete [29,30].

Due to the porous structure and water absorption characteristics of clay minerals, clay bricks exhibit strong water absorption. For clay bricks without wet processing, the CSA cement repair mortar easily falls off. Therefore, wet processing is indispensable. Under tension, the CSA cement repair mortar was pulled away from the clay bricks. For CSA cement repair mortar with 1.5% PVA, more brick was peeled from the surface of the bricks. This demonstrates that PVA could improve the bonding strength between the bricks and CSA cement repair mortar.

### 3.3. Hydration

#### 3.3.1. XRD Analysis of Hydration Products within 60 min

**Figure 8:**
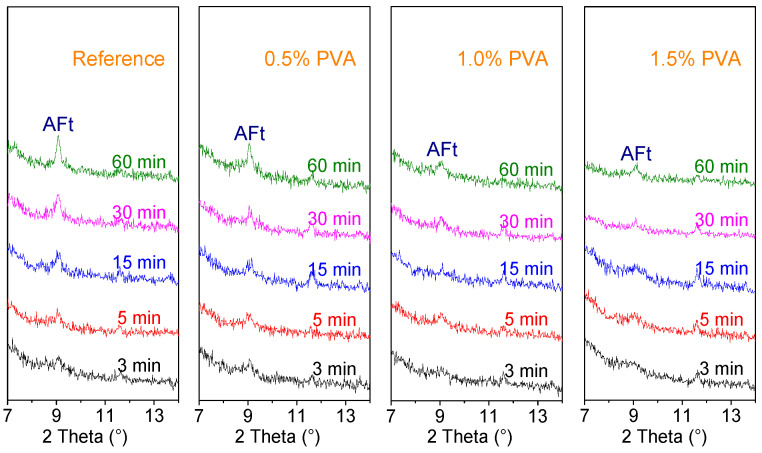
XRD patterns of the hydration products of the Reference, 0.5% PVA, 1.0% PVA, and 1.5% PVA samples at hydration ages of 3 min, 5 min, 15 min, 30 min, and 60 min.

After mixing with water, the PVA molecules in water concentrate at the surface of the cement particles, which inevitably affects the early age hydration of CSA cement and consequently influences the setting time of the cement. To study the influence of PVA on the hydration of CSA cement within 1 h, the hydration products of the Reference sample and the CSA cement samples with 0.5% PVA, 1.0% PVA and 1.5% PVA at the hydration ages of 3 min, 5 min, 15 min, 30 min, and 60 min were submitted to XRD analysis; the XRD patterns are shown in Figure 8.

For the Reference sample and the CSA cement samples with 0.5% PVA and 1.0% PVA, the diffraction peak of AFt can be observed at 3 min, while the AFt diffraction peak of the CSA cement with 1.5% PVA is almost undetectable. With prolonged hydration time, the diffraction peak intensity of AFt for the Reference increases obviously, while the diffraction peak intensity of AFt for the CSA cement with 0.5% PVA, 1.0% PVA, and 1.5% PVA increases slightly. All of this demonstrates that the presence of PVA could inhibit the early age hydration of C_4_A_3_$ and the precipitation of AFt. Therefore, the setting time of PVA-modified CSA cement is prolonged.

#### 3.3.2. Hydration Heat

**Figure 9:**
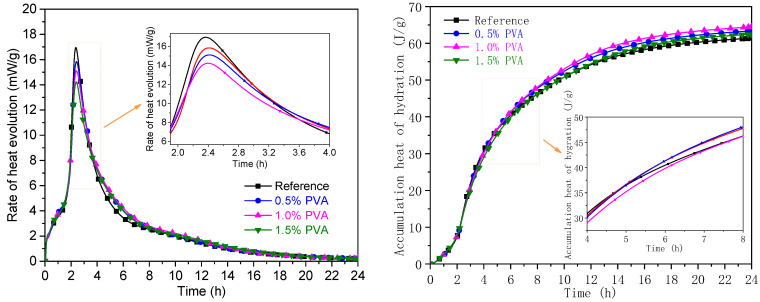
Heat flow and cumulative heat flow of the Reference sample and CSA cement samples with 0.5% PVA, 1.0% PVA, and 1.5% PVA within 24 h.

For CSA cement, the heat release at early hydration age is mainly ascribed to the hydration of C_4_A_3_$ [39,40,41]. Therefore, the hydration heat flow and cumulative heat can reflect the hydration rate and hydration degree of C_4_A_3_$. Figure 9 shows the heat flow and cumulative heat of the Reference sample and the CSA cement samples with 0.5% PVA, 1.0% PVA, and 1.5% PVA within 24 h.

The heat flow curves of the Reference sample and the CSA cement with 0.5% PVA, 1.0% PVA, and 1.5% PVA can be characterized by one exothermic peak. At the hydration age of 2.4 h, the heat flow reaches a maximum. With increasing PVA content in the CSA cement, the heat flow decreases obviously, which demonstrates that PVA could inhibit the early age hydration of C_4_A_3_$.

At the hydration age of 6 h, the cumulative heat for the CSA cement samples with 0.5% PVA and 1.0% PVA exceeds that for the Reference, which demonstrates that the hydration of C_4_A_3_$ is promoted. It indicates that the decrease in compressive strength at 6 h is caused by insufficient formation of the PVA film, which weakens the bonding strength between the hydration products. At the hydration age of 24 h, the cumulative heat for the CSA cement samples with 0.5% PVA, 1.0% PVA, and 1.5% PVA exceeds that for the Reference, which demonstrates a relatively higher hydration degree for C_4_A_3_$. This result is in accordance with the compressive strength development at the hydration age of 1 d.

#### 3.3.3. XRD Analysis of Hydration Products at 6 h, 1 d, and 3 d

**Figure 10:**
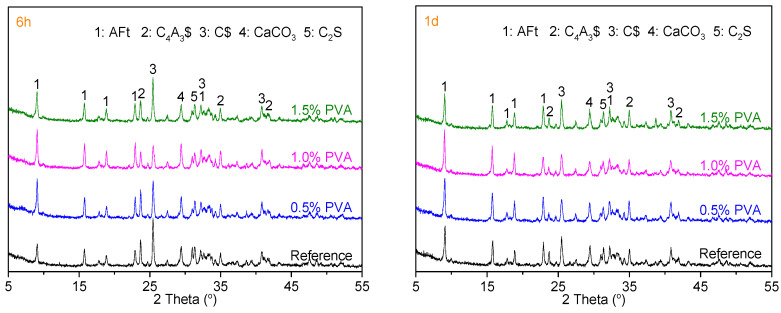

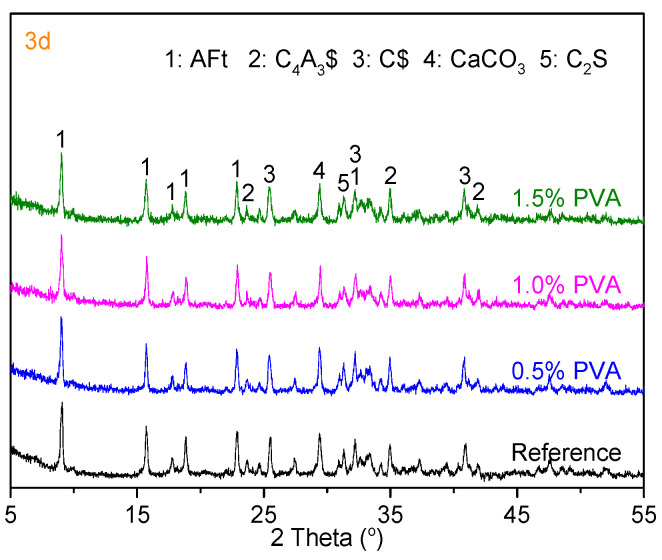
XRD patterns of Reference, 0.5% PVA, 1.0% PVA, and 1.5% PVA samples at the hydration ages of 6 h, 1 d, and 3 d.

For CSA cement, the strength gain at 6 h, 1 d, and 3 d is mainly ascribed to the hydration of C_4_A_3_$ to form AFt/AFm and aluminum hydroxide gel [40]. To study the influence of PVA on the formation of the hydration products, the hydration products of the Reference sample and the CSA cement samples with 0.5% PVA, 1.0% PVA, and 1.5% PVA at the hydration ages of 6 h, 1 d, and 3 d were submitted to XRD analysis; the XRD patterns are shown in Figure 10.

At early hydration age, the main hydration products of AFt and amorphous Al(OH)_3_ are formed by the hydration of C_4_A_3_$ and C$, respectively [39,40]. At the hydration age of 6 h, for the CSA cement samples with 0%, 0.5%, and 1.0% PVA, the diffraction intensities of AFt and CaSO_4_ present increasing and decreasing trends, respectively. When the PVA content of CSA cement is 1.5%, the diffraction peak intensity of AFt decreases and the diffraction peak intensity of CaSO_4_ increases. Therefore, from the perspective of promoting the hydration of C_4_A_3_$ at 6 h, the optimum PVA content should be below 1.0%. At the hydration age of 1 d, similar results were obtained, which is in accordance with the result for the cumulative heat at 1 d. At the hydration age of 3 d, the diffraction peak intensities for C_4_A_3_$, C$, and AFt of the Reference sample and the CSA cement samples with PVA are almost the same, which demonstrates that PVA does not affect the hydration of C_4_A_3_$ at 3 d.

#### 3.3.4. Thermal Analysis

**Figure 11:**
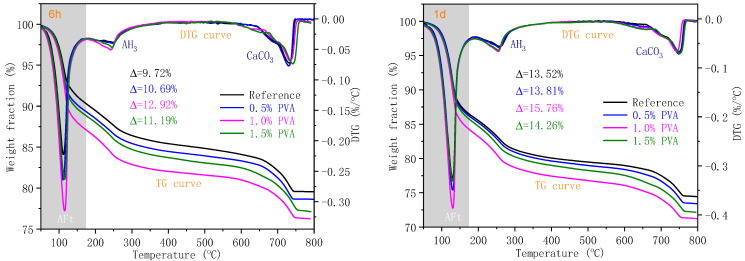

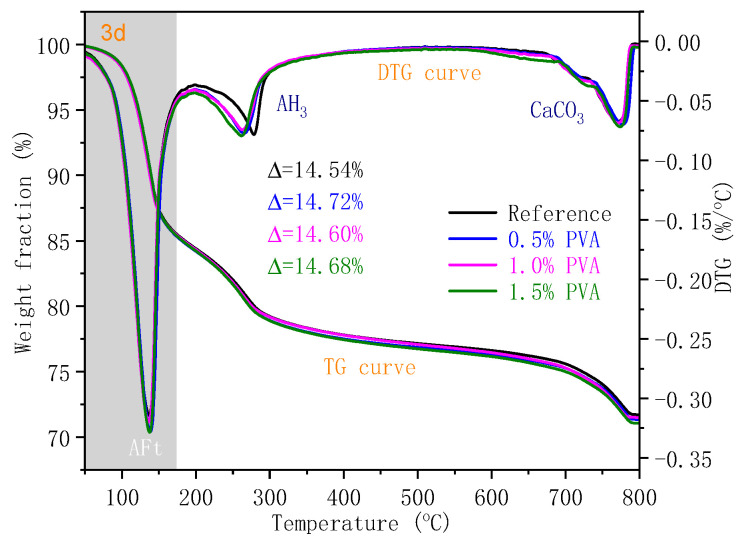
TG and DTG curves of the hydration products of the Reference, 0.5% PVA, 1.0% PVA, and 1.5% PVA samples at 6 h, 1 d, and 3 d.

To demonstrate the influence of PVA on the early age hydration of CSA cement, the hydration products of the Reference sample and the CSA cement samples with 0.5% PVA, 1.0% PVA, and 1.5% PVA at 6 h, 1 d, and 3 d were submitted to TG-DTG analysis; the curves are shown in Figure 11.

For CSA cement, the weight loss within the temperature ranges of 50~175 °C, 200~300 °C, and 600~800 °C is mainly ascribed to the dehydration of AFt, AFm (3CaO·Al_2_O_3_·CaSO_4_·12H_2_O), and CaSO_4_·2H_2_O; dehydration of Al(OH)_3_; and decomposition of CaCO_3_, respectively [39,42]. Figure 10 demonstrates that CaSO_4_ is adequate within the hydration age range of 0–3 d. Therefore, AFt is the main hydration product, and the AFt content could indicate the hydration degree of C_4_A_3_$.

At the hydration ages of 6 h and 1 d, the weight loss for the dehydration of AFt increases within the PVA dosage range of 0~1.0% and decreases at the PVA dosage of 1.5%. This result is in accordance with the results of XRD and hydration heat analysis. At the hydration age of 3 d, the weight loss values of the Reference sample and the CSA cement samples with 0.5% PVA, 1.0% PVA, and 1.5% PVA caused by the dehydration of AFt are almost the same, which demonstrates that PVA does not affect the hydration of C_4_A_3_$ at the hydration age of 3 d. The result is in accordance with the XRD results.

#### 3.3.5. Ion Concentration and pH of the Pore Solution and SI of AFt

**Figure 12:**
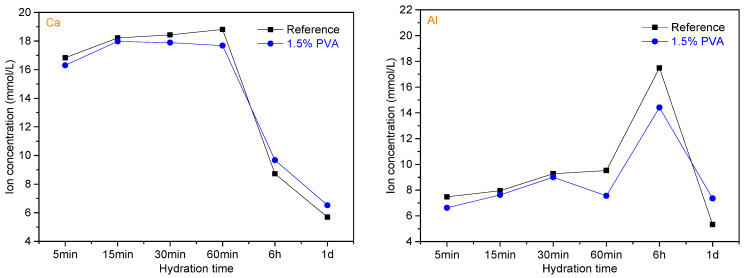

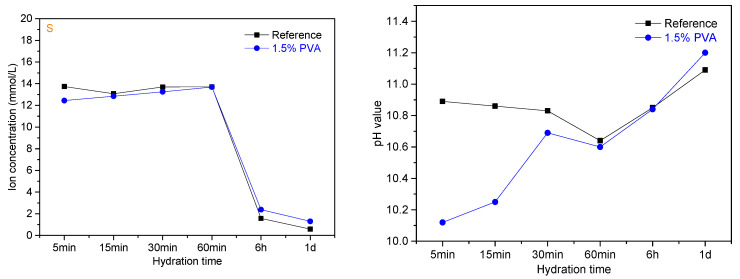
Ion (Ca, Al, and S) concentrations and pH values of the leachates of the Reference sample and the CSA cement sample with 1.5% PVA.

The PVA molecules are easily absorbed on the surface of the cement particles, thereby hindering the dissolution of the cement minerals and the formation of the hydration products [26,43]. To provide a clear understanding of the mechanism of the influence of PVA on the hydration of CSA cement, the leachates of slurry prepared from the Reference sample and the CSA cement sample with 1.5% PVA were submitted to ICP analysis; the ion (Ca, Al, and S) concentrations and pH values of the leachates are shown in Figure 12.

Within the hydration age of 60 min, the ion concentrations of Ca, Al, and S for the CSA cement with 1.5% PVA are obviously lower than those for the Reference, which proves that PVA could inhibit the early age dissolution of C_4_A_3_$. At the hydration age of 6 h, the ion concentrations of Ca and S for the CSA cement with 1.5% PVA are higher than those for the Reference, while the ion concentration of Al is relatively lower. This demonstrates that dissolution rates of C_4_A_3_$ and CaSO_4_ are hindered and promoted, respectively. At the hydration age of 1 d, the ion concentrations of Ca, Al, and S for the CSA cement with 1.5% PVA are obviously higher than those for the Reference, which proves that the dissolution of C_4_A_3_$ and CaSO_4_ is promoted.

The pH value is an important factor affecting the early age formation of AFt [44]. Within the hydration age of 6 h, the pH value for the CSA cement with 1.5% PVA is much lower than that for the Reference. This is mainly assigned to the residual acetic acid and acetic acid ester in PVA neutralizing the alkaline substances generated by the dissolution of CSA cement particles. At the hydration age of 1 d, the pH value for 1.5% PVA is higher than that for the Reference.

**Figure 13:**
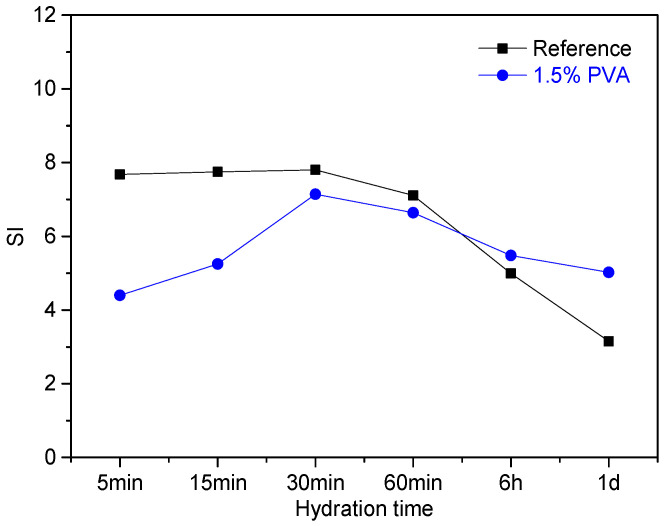
SI of Aft within the hydration age of 1 d.

For a solid phase, when the SI > 0, SI < 0, or SI = 0, it is considered to be supersaturated, unsaturated, or at equilibrium, respectively [45,46]. To study the influence of PVA on the precipitation of AFt, the SI of AFt within the hydration age range of 5 min to 1 d was calculated; the result is shown in Figure 13.

Within the hydration age range of 5~60 min, the SI of Aft for the CSA cement with 1.5% PVA is lower than that for the Reference. This is mainly due to the following reasons: (i) the PVA molecules are absorbed on the surface of the cement particles, which hinders the dissolution of C_4_A_3_$ and thereby inhibits the precipitation of Aft; (ii) PVA contains a small amount of residual acetic acid and polyvinyl acetate, which lowers the pH value of the leachates and adversely affects the formation of Aft. This well explains why the setting time of CSA cement is prolonged in the presence of PVA. At the hydration age of 1 d, the SI of Aft for 1.5% PVA is higher than that for the Reference. This demonstrates that the formation of Aft is promoted, which is in accordance with the results of hydration heat and XRD analysis.

#### 3.3.6. XPS Analysis

**Figure 14:**
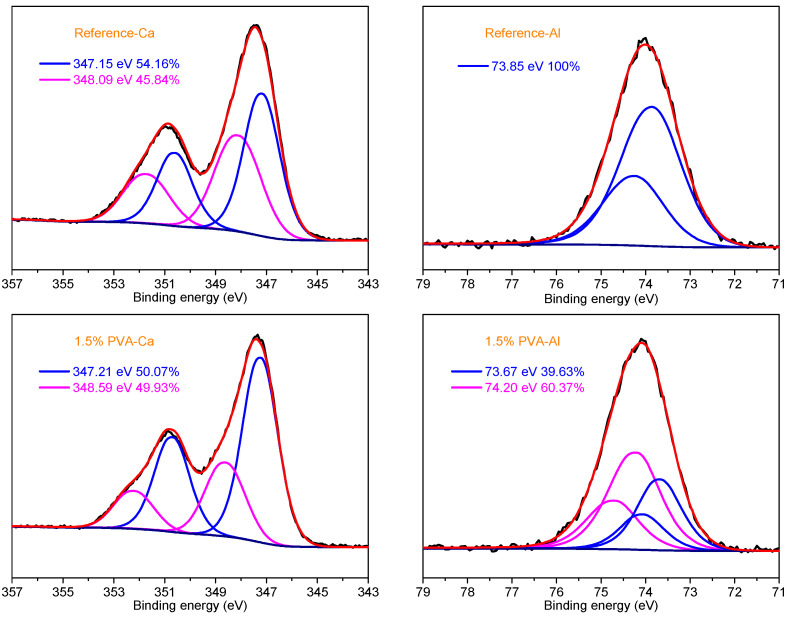
Fitted XPS spectra of Ca 2p and Al 2p for the hydration products of the Reference sample and the CSA cement sample with 1.5% PVA at the hydration age of 28 d.

In the hydration system of Portland cement, calcium aluminate cement, and magnesium phosphate cement, PVA could react with Ca, Al, and Mg ions to form the chemical bonds C-O-Ca, C-O-Al, and C-O-Mg, which could join the repair mortar and the old substrate to improve the bonding strength [26,31,32]. To provide a clear understanding of the mechanism of the improvement of the bonding strength between the CSA repair mortar and the clay bricks, the hydration products of the Reference sample and the CSA cement sample with 1.5% PVA at the hydration age of 28 d were submitted to XPS analysis; the fitted XPS spectra of Ca 2p and Al 2p are shown in Figure 14.

All the Ca 2p spectra exhibit two main peaks at 347.39 eV and 347.33 eV. The shape and size of the Ca 2p peaks are almost the same, which demonstrates that no new material is generated and Ca does not react with PVA. For the Al 2p spectra, the peak at 73.09 eV belongs to the Al in the hydration products and C_4_A_3_$ with tetrahedral coordination [47]. For the Al 2p spectrum of the CSA cement with 1.5% PVA, the peak located at 75.64 eV confirms the existence of the C-O-Al chemical bond, as presented in the hydration system of calcium aluminate cement [32,48].

**Figure 15:**
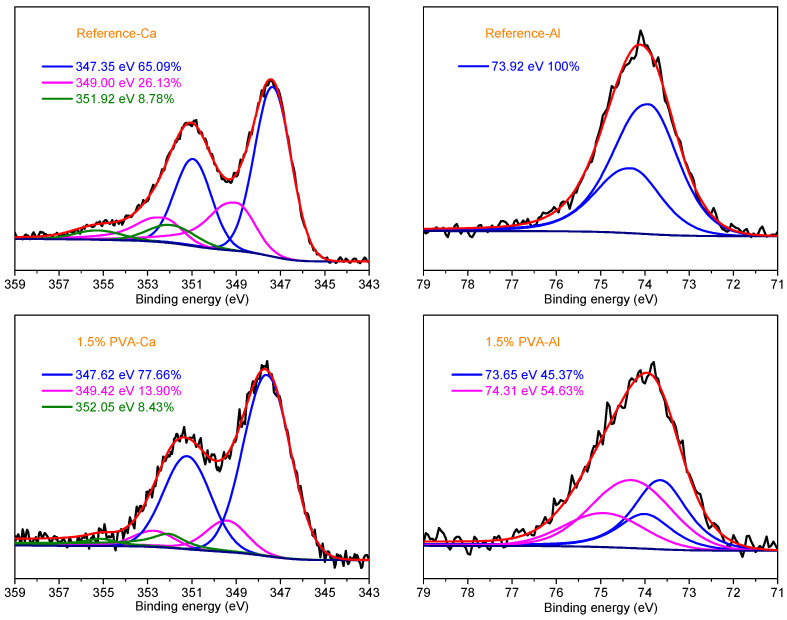
Fitted XPS spectra of Ca 2p and Al 2p of the Reference sample and the brick powder with 1.5% PVA at the hydration age of 28 d.

Figure 15 shows the fitted XPS spectra of Ca 2p and Al 2p of the Reference sample and the clay brick powder with 1.5% PVA at the hydration age of 28 d. The Al ions in the clay bricks could also react with PVA to form the chemical bond C-O-Al. Therefore, the combination of the CSA cement repair mortar and the clay bricks is tighter and the bonding strength is improved in the presence of PVA.

## 4. Conclusions

In this study, PVA and CSA cement were used to prepare a repair mortar for the restoration of the walls of buildings built with bricks. The preparation, hydration, and properties of PVA-modified CSA cement repair mortar were studied. The mechanism by which PVA improves the bonding strength was also discussed. The following conclusions can be drawn from the above study:

(1) PVA can inhibit the early age dissolution of C_4_A_3_$ and the precipitation of AFt within the hydration age range of 0~60 min. Therefore, the setting time of CSA cement is prolonged with the presence of PVA.
(2) PVA lowers the mechanical strength at 6 h. This is mainly due to PVA inhibiting the hydration of C_4_A_3_$ within the hydration age of 6 h. Besides this, at the hydration age of 6 h, PVA fails to form a film and is evenly distributed among hydration products, which weakens the bonding strength between the hydration products.
(3) At the hydration ages of 1 d, 3 d, 7 d, and 28 d, the mechanical strength of CSA cement repair mortar with 0.5% PVA, 1.0% PVA, and 1.5% PVA exceeds that of the Reference. This is due to the hydroxyl group in PVA reacting with the Al ions in the hydration products, which improves the linkage between the hydration products; PVA could also promote the hydration of C_4_A_3_$ and the formation of AFt within the hydration age range of 6 h to 3 d. Besides this, PVA forms a film filling the cracks of the hardened CSA cement repair mortar and lowers the porosity.
(4) The Al ions in both the hydration products of CSA cement and the clay bricks could react with the hydroxyl group of PVA and form C-O-Al chemical bonds. A tighter combination of the CSA cement repair mortar and the bricks forms, and the bonding strength is improved. Therefore, PVA-modified CSA cement is suitable for the restoration of wall structures built with clay bricks.

## Figures and Tables

**Table 1:** Chemical composition, expressed in oxides, of CSA.

Oxides	CaO	SO_3_	Al_2_O_3_	SiO_2_	MgO	Fe_2_O_3_	TiO_2_	K_2_O	Na_2_O	Others	LOI
Weight fraction/%	45.98	17.3	16.8	8.92	2.56	2.16	0.75	0.23	0.16	0.51	5.65

**Table 2:** Physical properties of the sand.

Physical Properties	Value
Clay content (%)	3.33
Apparent density (kg/m^3^)	1868
Accumulation density (kg/m^3^)	1459
Void fraction (%)	22
Moisture content (%)	0.36
Hardness	Ⅱ
MB value (g/kg)	2.30
Stone powder content (%)	2.67
Dry water absorption of saturated surface (%)	0.87

**Table 3:** Physical and chemical properties of PVA.

Properties	pH Value	Viscosity (mPa·s)	Alcoholysis Degree (%)	Volatile Content (%)	Ash Content (%)	Purity (%)	Average Molecular Weight (g/mol)
Value	5.0~7.0	4.5~6.5	87.0~89.0	≤5.0	≤0.5	≥95.0	16,000–20,000

**Table 4:** Composition of CSA cement repair mortars.

Sample	CSA Cement (g)	Sand (g)	PVA (%)	Water (g)	Polycarboxylate Superplasticizer (%)	Citric Acid (%)	Tributyl Phosphate (%)	Fluidity (mm)
Reference	450	1350	0	237	0.30	0.2	0.2	169.5
0.5% PVA	450	1350	0.5	237	0.40	0.2	0.2	170.0
1.0% PVA	450	1350	1.0	237	0.55	0.2	0.2	169.5
1.5% PVA	450	1350	1.5	237	0.80	0.2	0.2	170.0
2.0% PVA	450	1350	2.0	237	2.35	0.2	0.2	170.5

## Data Availability

The data presented in this study are available on request from the corresponding author.

## References

[1] Liao W.Z., Wang H.W., Li M., Ma C., Wang B. (2019). Large scale experimental study on bond behavior between polymer modified cement mortar layer and concrete. Constr. Build. Mater..

[2] Aattache A., Soltani R. (2020). Durability-related properties of early-age and long-term resistant laboratory elaborated polymer-based repairing mortars. Constr. Build. Mater..

[3] Rashid K., Ueda T., Zhang D., Miyaguchi K., Nakai H. (2015). Experimental and analytical investigations on the behavior of interface between concrete and polymer cement mortar under hygrothermal conditions. Constr. Build. Mater..

[4] Guo S.Y., Zhang X., Chen J.Z., Mou B., Shang H.S., Wang P., Zhang L., Ren J. (2020). Mechanical and interface bonding properties of epoxy resin reinforced Portland cement repairing mortar. Constr. Build. Mater..

[5] Morgan D.R. (1996). Compatibility of concrete repair materials and systems. Constr. Build. Mater..

[6] Al-Zahrani M.M., Maslehuddin M., Al-Dulaijan S.U., Ibrahim M. (2003). Mechanical properties and durability characteristics of polymer- and cement-based repair materials. Cem. Concr. Compos..

[7] Burris L.E., Kurtis K.E. (2018). Influence of set retarding admixtures on calcium sulphoaluminate cement hydration and property development. Cem. Concr. Res..

[8] Thong C.C., Teo D.C.L., Ng C.K. (2016). Application of polyvinyl alcohol (PVA) in cement-based composite materials: A review of its engineering properties and microstructure behavior. Constr. Build. Mater..

[9] Luo J.L., Li Q.Y., Zhao T.J., Gao S., Sun S. (2013). Bonding and toughness properties of PVA fibre reinforced aqueous epoxy resin cement repairing mortar. Constr. Build. Mater..

[10] Medeiros M.H.F., Helene P., Selmo S. (2009). Influence of EVA and acrylate polymers on some mechanical properties of cementitious repairing mortars. Constr. Build. Mater..

[11] Betioli A.M., Gleize P.J.P., John V.M., Pileggi R.G. (2012). Effect of EVA on the fresh properties of cement paste. Cem. Concr. Compos..

[12] Shi C., Zou X.W., Wang P. (2018). Influences of ethylene-vinyl acetate and methylcellulose on the properties of calcium sulphoaluminate cement. Constr. Build. Mater..

[13] Silva D.A., John V.M., Ribeiro J.L.D., Roman H.R. (2001). Pore size distribution of hydrated cement pastes modified with polymers. Cem. Concr. Res..

[14] Aggarwal L.K., Thapliyal P.C., Karade S.R. (2007). Properties of polymer-modified mortars using epoxy and acrylic emulsions. Constr. Build. Mater..

[15] Xiang Q., Xiao F.P. (2020). Applications of epoxy materials in pavement engineering. Constr. Build. Mater..

[16] Wang R., Wang P.M., Li X.G. (2005). Physical and mechanical properties of styrene-butadiene rubber emulsion modified cement mortars. Cem. Concr. Res..

[17] Rossignolo J.A., Agnesini M.V.C. (2002). Mechanical properties of polymer-modified lightweight aggregate concrete. Cem. Concr. Res..

[18] Assaad J.J. (2018). Development and use of polymer-modified cement for adhesive and repair applications. Constr. Build. Mater..

[19] Tian Y., Jin X.Y., Jin N.G., Zhao R., Li Z.J., Ma H.Y. (2013). Research on the microstructure formation of polyacrylate latex modified mortars. Constr. Build. Mater..

[20] Li Y.L., Li W.G., Deng D.H., Wang K., Duan W.H. (2018). Reinforcement effects of polyvinyl alcohol and polypropylene fibers on flexural behaviors of sulphoaluminate cement matrices. Cem. Concr. Compos..

[21] Li L., Wang R., Lu Q.Y. (2018). Influence of polymer latex on the setting time, mechanical properties and durability of calcium sulphoaluminate cement mortar. Constr. Build. Mater..

[22] Rashid K., Wang Y., Ueda T. (2019). Influence of continuous and cyclic temperature durations on the performance of polymer cement mortar and its composite with concrete. Compos. Struct..

[23] Wang R., Li X.G., Wang P.M. (2006). Influence of polymer on cement hydration in SBR-modified cement pastes. Cem. Concr. Res..

[24] Zheng Z., Li Y.X., Ma X., Zhu X., Li S. (2019). High density and high strength cement-based mortar by modification with epoxy resin emulsion. Constr. Build. Mater..

[25] Bourguib A., Ghorbel E., Cristofol L., Dhaoui W. (2017). Effects of recycled sand on the properties and durability of polymer and cement based mortars. Constr. Build. Mater..

[26] Xie Y., Lin X., Li H., Ji T. (2020). Effect of polyvinyl alcohol powder on the bonding mechanism of a new magnesium phosphate cement mortar. Constr. Build. Mater..

[27] Wang M., Wang R., Zheng S., Farhan S., Yao H., Jiang H. (2015). Research on the chemical mechanism in the polyacrylate latex modified cement system. Cem. Concr. Res..

[28] Wang M., Wang R., Yao H., Farhan S., Zheng S., Wang Z., Du C., Jiang H. (2016). Research on the mechanism of polymer latex modified cement. Constr. Build. Mater..

[29] Kim J.H., Robertson R.E. (1998). Effects of polyvinyl alcohol on aggregate-paste bond strength and the interfacial transition zone. Adv. Cem. Based Mater..

[30] Allahverdi A., Kianpur K., Moghbeli M.R. (2010). Effect of polyvinyl alcohol on flexural strength and some important physical properties of Portland cement paste. Iran. J. Mater. Sci. Eng..

[31] Ekincioglua O., Ozkul M.H., Struble L.J., Patachia S. (2012). Optimization of material characteristics of macro-defect free cement. Cem. Concr. Compos..

[32] Kalina L., Másilko J., Koplík J., Šoukal F. (2014). XPS characterization of polymer-monocalcium aluminate interface. Cem. Concr. Res..

[33] Knapen E., Gemert D.V. (2009). Cement hydration and microstructure formation in the presence of water-soluble polymers. Cem. Concr. Res..

[34] Afridi M.U.K., Ohama Y., Demura K., Iqbal M.Z. (2003). Development of polymer films by the coalescence of polymer particles in powdered and aqueous polymer-modified mortars. Cem. Concr. Res..

[35] Knapen E., Gemert D.V. (2015). Polymer film formation in cement mortars modified with water-soluble polymers. Cem. Concr. Compos..

[36] Péra J., Ambroise J. (2004). New applications of calcium sulphoaluminate cement. Cem. Concr. Res..

[37] Wang R., Wang P.M. (2011). Action of redispersible vinyl acetate and versatate copolymer powder in cement mortar. Constr. Build. Mater..

[38] Kim J.H., Robertson R.E. (1997). Prevention of air void formation in polymer-modified cement mortar by pre-wetting. Cem. Concr. Res..

[39] Winnefeld F., Lothenbach B. (2010). Hydration of calcium sulphoaluminate cements-Experimental findings and thermodynamic modelling. Cem. Concr. Res..

[40] Huang Y.B., Qian J.S., Liu C., Liu N., Shen Y., Ma Y., Sun H., Fan Y. (2017). Influence of phosphorus impurities on the performances of calcium sulphoaluminate cement. Constr. Build. Mater..

[41] Huang Y.B., Qian J.S., Liang J., Liu N., Li F., Shen Y. (2016). Characterization and calorimetric study of early-age hydration behaviors of synthetic ye’elimite doped with the impurities in phosphogypsum. J. Therm. Anal. Calorim..

[42] Zajac M., Skocek J., Bullerjahn F., Haha M.B. (2016). Effect of retarders on the early hydration of calcium-sulpho-aluminate (CSA) type cements. Cem. Concr. Res..

[43] Pique T.M., Balzamo H., Vazquez A. (2011). Evaluation of the hydration of portland cement modified with polyvinyl alcohol and nano clay. Key Eng. Mater..

[44] Qian J.S., Yu J.C., Sun H.Q., Ma Y. (2017). Formation and function of ettringite in cement hydrates. J. Chin. Ceram. Soc..

[45] Kang X.J., Zhu X.H., Liu J.P., Shu X., Huang Y., Qian J. (2020). Dissolution and precipitation behaviours of graphene oxide/tricalcium silicate composites. Compos. Part B: Engeer..

[46] Zhu X., Zhang M., Yang Y., Yang K., Wu F., Li Q., Yu L., Yang C., Basheer M. (2019). Understanding the aqueous phase of alkali-activated slag paste under bath-curing. Adv. Cement. Res..

[47] Sandrin L., Sacher E. (1998). X-ray photoelectron spectroscopy studies of the evaporated aluminum / corona-treated polyethylene terephthalate interface. Appl. Surf. Sci..

[48] Bou M., Martin J.M., Monge T.L. (1991). Chemistry of the interface between aluminium and polyethyleneterephthalate by XPS. Appl. Surf. Sci..

